# Effects of Relaxing Music on Healthy Sleep

**DOI:** 10.1038/s41598-019-45608-y

**Published:** 2019-06-24

**Authors:** Maren Jasmin Cordi, Sandra Ackermann, Björn Rasch

**Affiliations:** 1 University of Fribourg, Department of Psychology, Division of Cognitive Biopsychology and Methods, Fribourg, Switzerland; 2 Sleep & Health Zürich, University of Zurich, Zurich, Switzerland

**Keywords:** Human behaviour, Quality of life

## Abstract

Sleep is vital for human health and wellbeing, and sleep disturbances are comorbid to many mental and physiological disorders. Music consistently improves subjective sleep quality, whereas results for objective sleep parameters diverge. These inconsistencies might be due to inter-individual differences. Here, 27 female subjects listened to either music or a control text before a 90 minutes nap in a within-subjects design. We show that music improved subjective sleep quality as compared to the text condition. In all participants, music resulted in a reduced amount of sleep stage N1 during the nap. In addition, music significantly increased the amount of slow-wave sleep (SWS) and increased the low/high frequency power ratio. However, these effects occurred only in participants with a low suggestibility index. We conclude that listening to music before a nap can improve subjective and objective sleep parameters in some participants.

## Introduction

Sleep plays an important role for maintaining physical and mental health^1–5^, and is critical for general well-being^6,7^. However, sleep disturbances are highly common in our society^8^, with increased prevalence in ageing^9^ as well as among people at risk of or suffering from a psychiatric disorder^10^. The use of sleep enhancing medicine is problematic, as its effectiveness decreases across time and may lead to addiction. Consequently, researchers need to empirically validate the effectiveness of non-pharmacological and easy to implement tools to support healthy sleep.

Listening to music is a widely used tool to improve sleep. In an online survey in a general population 62% (out of n = 651 respondents) stated to have at least once used music to help them sleep^11^. In a survey in over 500 patients with sleep disorders, over 50% reported to use music as sleep aid^12^. In a meta-analysis based on six included studies in a total of 314 patients, Jespersen and colleagues reported that music helped to improve subjective sleep quality in insomnia patients^13^. Similarly, sedative music while resting could effectively improve subjectively rated sleep in patients with sleep complaints^14^. In 20 included trials, another meta-analysis showed positive effects of music based interventions before bedtime on PSQI scores, sleep onset latency and sleep efficiency in patients suffering from primary insomnia^15^. Positive effects of listening to music on subjectively evaluated sleep were found in toddlers when listening throughout the naptime, preschool children and young adults listening 45 minutes at naptime and/or bedtime^16–18^. Also in older women, two further studies reported shorter sleep onset time, less nocturnal awakenings and better sleep quality and satisfaction as measured with a questionnaire and sleep logs when listening to music when going to bed^19,20^. In sum, the positive effects of listening to music on subjective ratings of sleep quality are well established across different age groups including both healthy participants and patients.

In contrast to subjective sleep quality, empirical findings on the effects of music on objectively measured sleep are scarce and inconsistent. For example, Lazic and Ogilvie^21^ did not find differences in polysomnographic measures when subjects in a within design either listened to music, tones or neither tones nor music after lights off until continuous sleep was observed. Similarly, Chang *et al*.^22^ did not observe any positive effects of music playing when lying in bed on objective measures of sleep onset, total sleep time, sleep interruption and sleep efficiency, in spite of positive effects of music on subjective sleep quality. Only Chen *et al*.^23^ reported effects of music on objective sleep parameters: One hour of listening to music after subjects went to bed significantly decreased the amount of stage N2 sleep, and increased deep SWS only in a subgroup of participants with long sleep latencies. However, further, more fine grained analyses of sleep-related oscillations are missing in this study. This paper points towards the possibilities that the effects of music on objective sleep parameters might depend on certain individual differences.

One possible factor being involved in individual differences in the effects of non-pharmacological interventions on objective sleep may be suggestibility. Suggestibility describes the ability of a person to respond to suggestions in terms of perceptual, cognitive, neural and bodily processes^24^. Suggestibility accounts for strong individual differences in the effects of hypnotic interventions, but also outside of hypnosis, like for instance “placebo” effects^25^ (but see^26^). Also, a suggestion to not attribute meaning to Stroop words reduced the Stroop effect in highly suggestible subjects irrespective of whether a hypnotic state was established before or not^27^. It was even shown that suggestions impact on memories, beliefs and behaviors^28^. In a series of studies^29^, we have shown that hypnotic suggestion to sleep deeper increases deep SWS and slow wave activity (SWA) in high suggestible healthy participants. No enhancement of SWS was observed for low suggestible females. A similar pattern of results occurred in a conceptual replication of this study in elderly women^30^. As we here applied the same study design as in those previous experiments, we were interested if the effect of music on sleep also depends on expectations and self-suggestions. We expect that the effect of music on objective sleep parameters and sleep related oscillations in the electroencephalogram (EEG) is stronger in high vs. low suggestible participants.

In the current study we tested if listening to music as compared to a spoken text before a midday nap improves sleep quality in high vs. low suggestible females. We measured sleep objectively using polysomnography. The experimental design was identical to our previous studies on the effects of hypnotic suggestions on sleep, except that a musical piece instead of the standardized hypnotic suggestion was used. The musical piece was composed by Dr. Lee Bartel for promoting sleep, called “Drifting into Delta”track^2,31^. It was pre-selected in a pilot study due to its best effects on subjective ratings of sleep quality. As it is supposed to induce deep sleep through its frequencies of 0.01–2 Hz we also analyzed brain activity during listening to the sound. To foster the role of subjective expectations and beliefs, subjects were explicitly informed that the music composition used was specifically designed to deepen sleep. Consequently we also assumed to find increased low and reduced high frequency EEG activity during the nap following the music condition. In order to test possible consequences of altered sleep on cognitive performance, we additionally introduced memory tests before and after the nap.

## Results

### Subjective sleep measures

As expected from previous studies, subjects reported better subjective sleep after music (3.69 ± 0.16, scale from 1 to 5) as compared to the control text (3.28 ± 0.18) as indicated by a significant main effect of sound on the dependent variable subjective sleep quality (*F*(1, 24) = 4.36, *p* = 0.048, eta^2^ = 0.15). The interaction with suggestibility did not reach significance (*p* = 0.15).

### Objective sleep measures

**Figure 1:**
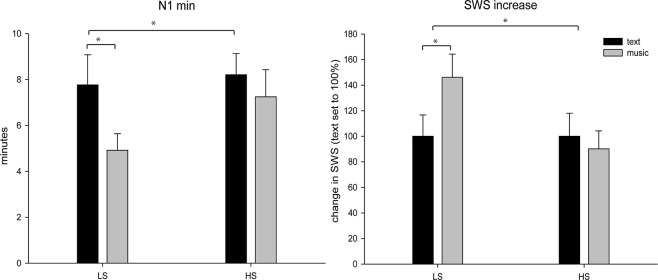
Effect of music on sleep stages. (**A**) Both groups benefitted from reduced minutes spent in N1 after music compared to text condition. The within-group comparison was significant only for low suggestible subjects. (**B**) The interaction between sound and suggestibility in SWS increase was driven by low suggestibles showing a higher increase in SWS after music compared to text while high suggestibles did not benefit from music. The x-axes distinguish low (LS) and high suggestible (HS) subjects. The graphs show the values for N1 minutes and SWS increase in percent in the text (black bar) and music condition (grey bar). Asterisks indicate *p* values ≤ 0.05. Mean + /− standard errors of the mean (SEM) are displayed.

For objective measures of sleep architecture, only one parameter revealed a significant effect: Listening to music before the 90 min nap decreased the time spent in N1 sleep (6.13 ± 0.73 min) as compared to the control condition (8.00 ± 0.78 min, main effect sound: *F*(1, 25) = 5.39, *p* = 0.029, eta^2^ = 0.18, see Fig. 1A). In all other sleep stages, the main effect of sound was non-significant (all *p* ≥ 0.15 see Table 1). Additionally, we observed a significant interaction between the type of sound and suggestibility on SWS increase (where the amount of SWS in the control condition was set to 100%): In low suggestibles, music increased the percentage of SWS by +46.18% (*p* = 0.035), whereas a non-significant reduction of SWS (−9.84%, *p* = 0.60) occurred in high suggestible participants (interaction *F*(1, 25) = 4.37, *p* = 0.047, eta^2^ = 0.15), See Fig. 1B. In absolute values, low suggestibles spent 31.35 ± 3.83 min in SWS after listening to music as compared to 20.58 ± 3.58 min after listening to the control text (*p* = 0.027). In contrast, no difference in time spent in SWS occurred for high suggestible participants (23.11 ± 3.50 min vs. 23.86 ± 4.27 min, for music vs. control condition, *p* = 0.89). Both results were not affected when considering that during listening to music, 9 low and 11 high suggestible subjects fell asleep while 4 low and 3 high suggestibles did not (all *p* ≥ 0.25 for this factor).A chi-square test comparing the number of subjects falling asleep and suggestibility was non-significant (Chi^2^(1) = 0.31, *p* = 0.58). During listening to the text, 11 low and 11 high suggestibles fell asleep, while 2 low and 3 high suggestibles did not (Chi^2^(1) = 0.69, *p* = 0.16).

**Table 1:** Sleep Parameters in high and low suggestible subjects.

N = 27	High suggestible (n = 14)		Low suggestible (n = 13)		P sound	P Sug-gestibility	P inter-action
	music	text	music	text			
wake min	0.64 ± 0.35	0.82 ± 0.43	2.73 ± 1.49	6.50 ± 2.76	0.16	**0.03**	0.20
N1 min	7.25 ± 1.18	8.21 ± 0.92	4.92 ± 0.72	7.77 ± 1.31	**0.029**	0.28	0.26
N2 min	43.14 ± 2.68	41.25 ± 4.21	30.89 ± 3.64	33.62 ± 2.82	0.87	**0.024**	0.36
SWS min	23.11 ± 3.50	23.86 ± 4.27	31.35 ± 3.83	20.58 ± 3.58	0.15	0.56	0.10
REM min	**8.18** ± **2.26**	**2.82** ± **1.47**	2.92 ± 1.70	6.27 ± 2.60	0.56	0.70	**0.018**
%SWS increase	90.16 ± 14.06	100.00 ± 18.06	**146.18** ± **18.17**	**100.00** ± **16.69**	0.19	0.17	**0.047**
Sleep latency	8.29 ± 1.77	11.50 ± 2.89	11.08 ± 2.36	12.31 ± 3.12	0.24	0.57	0.59
SWS latency	21.39 ± 3.39	17.14 ± 1.49	21.89 ± 5.31	24.23 ± 5.45	0.73	0.47	0.23
TST	82.43 ± 1.83	77.04 ± 3.15	72.89 ± 3.86	74.89 ± 3.86	0.54	0.22	0.18
Subj. sleep quality	3.99 ± 0.20	3.30 ± 0.25	3.39 ± 0.24	3.27 ± 0.25	**0.048**	0.27	0.15
Spindle density	3.50 ± 0.20	3.53 ± 0.29	4.38 ± 0.53	4.58 ± 0.59	0.40	0.11	0.54
Fast spindle density in Pz	1.76 ± 0.17	1.76 ± 0.22	2.18 ± 0.20	2.17 ± 0.21	0.94	0.13	0.96
Slow spindle density in Fz	2.41 ± 0.16	2.47 ± 0.23	3.02 ± 0.49	3.37 ± 0.60	0.07	0.18	0.20

*Note. TST = total sleep time (including wake, N1, N2, SWS, REM and movement time); % SWS increase: relative amount of SWS with SWS minutes in text condition set to 100%. Sleep latency is the duration between lights off and last N1 before consolidated N2. “Spindle density” (11–15 Hz) refers to average of the derivations Fz, Cz and Pz within 1 minute of NREM sleep (N2 + N3 sleep). Density in fast (13–15 Hz) and slow spindles (11–13 Hz) were measured in Pz and Fz, respectively. Bold letters indicate significant within-group differences (all *p* < 0.05) in comparisons in which the interaction in the ANOVA was significant.

Additionally, the interaction between sound and suggestibility for REM sleep was significant (*F*(1, 25) = 6.39, *p* = 0.018, eta^2^ = 0.20), as demonstrated in Table 1. However, only few participants had actually reached REM sleep (high suggestible: 9 vs. 4 participants after music vs. text, low suggestibles: 3 vs. 5 participants). Although the Chi-square for a difference from an equal distribution was not significant (*p* > 0.15), we refrained from further analyses of this finding due to the very low sample size in this analysis.

Please note that differences in total sleep time (see Table 1) might rather be a product of divergent sleep latencies as time in bed was limited to 90 minutes. In general, high suggestible participants spent more time in N2 sleep and were less time awake after sleep onset as compared to low suggestibles (both *p* = 0.05, see Table 1).

### Poweranalyses during non-REM (NREM) sleep

For a more fine grained analysis of the effects of music on sleep, we compared power of sleep-related brain oscillations during NREM sleep stages N2 and SWS between the music and the control condition. The analyses were conducted using repeated measure ANOVAs with within-subject factors FCP (frontal, central, parietal), hemisphere (right vs. left) and type of sound (music vs. text) and between-subjects factor suggestibility (high vs. low). Total power (i.e., 0.5–50 Hz) did not differ significantly between the two experimental sessions (*p* > 0.16). Therefore, the poweranalyses are based on the absolute muV values in the single frequency bands.

**Figure 2:**
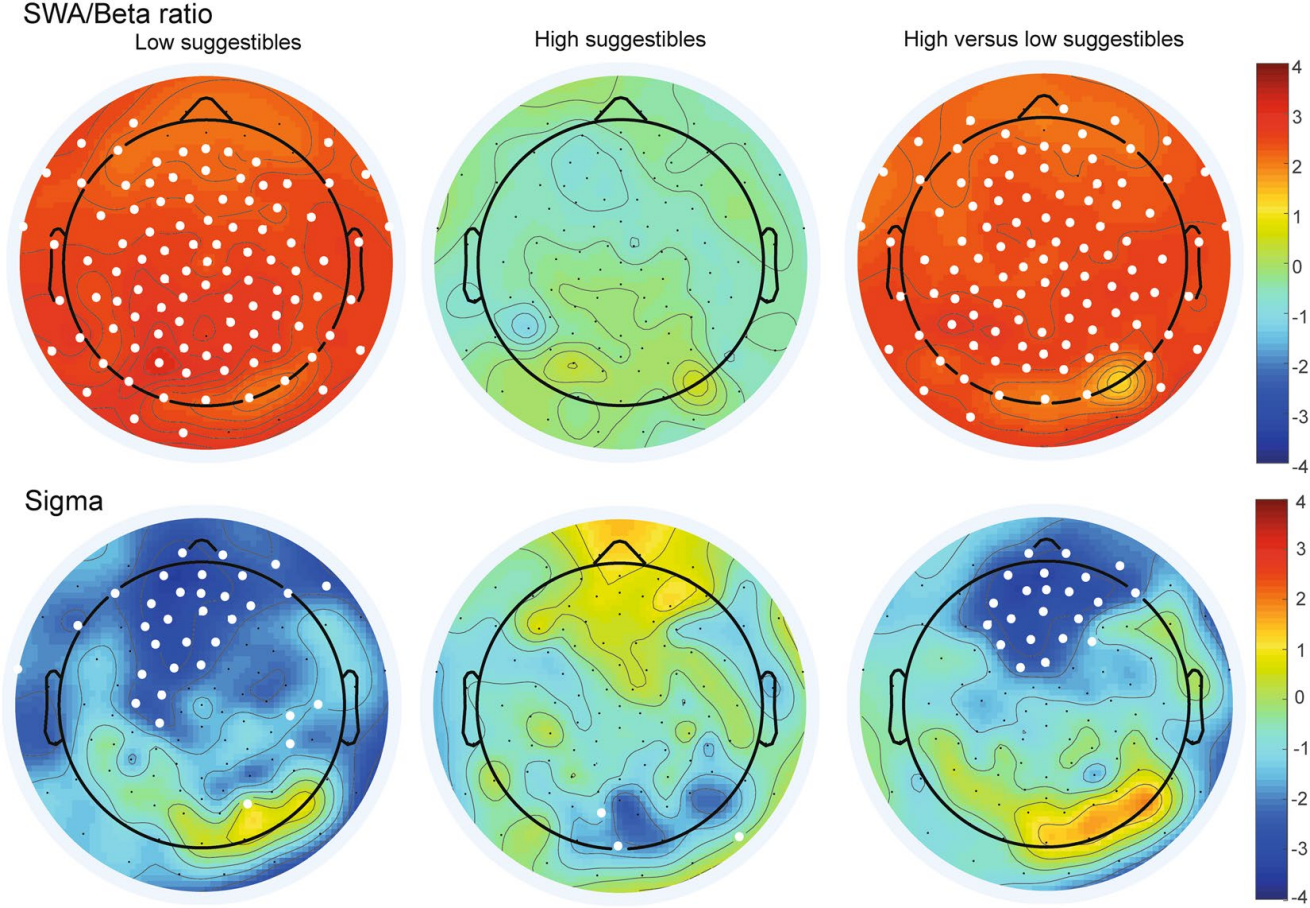
Power analyses during NREM sleep. Displays t-values of the analyses on SWA/beta Ratio (upper panel) and Sigma power (lower panel). The 1^st^ column shows comparisons on music versus text in low suggestibles, while higher values indicate higher power after music. The 2^nd^ column shows the results of the same analysis for high suggestibles. The 3^rd^ column indicates results of the group comparison high versus low suggestibles on the difference music – text. Positive values mean higher power value differences in low compared to high suggestibles. Significant electrodes (non-corrected for multiple comparisons) are indicated with white dots.

We focused on the ratio between low and high frequencies during NREM sleep, as this marker has been discussed as indicator for restorative sleep (SWA divided by beta activity, see^32^ and^33^). Higher values thus mean a higher proportion of low than high frequency power in the signal during NREM sleep and might be associated with more restorative sleep. Music significantly increased the low/high frequency ratio during sleep as compared to the control text only in low suggestible subjects (interaction: *F*(1, 25) = 5.72, *p* = 0.025, eta^2^ = 0.19). The post-hoc t-test showed a significantly higher ratio after music (154.07 ± 32.69) than after text (89.67 ± 15.46), *t*(12) = −2.45, *p* = 0.031), while this difference was non-significant in high suggestible subjects (*p* = 0.65). The effects were wide-spread across the whole scalp, see Fig. 2, upper panel, although the ANOVA indicated that this interaction was dependent on localization (three-way interaction between sound, suggestibility and FCP *F*(2, 50) = 3.90, *p* = 0.027, eta^2^ = 0.14). Following up on this revealed that in high suggestibles, FCP * sound was non-significant (*p* = 0.64), while the interaction was significant in low suggestible subjects (*F*(2, 24) = 3.40, *p* = 0.05, eta^2^ = 0.22. Here, all post-hoc tests were significant and showed a higher ratio in frontal (*t*(12) = 2.25, *p* = 0.044), central (*t*(12) = 2.57, *p* = 0.024) and parietal electrodes (*t*(12) = 2.71), *p* = 0.019). All other effects with sound or suggestibility in this ANOVA were non-significant (*p* > 0.06).

Analyzing SWA alone revealed a trend in the interaction between suggestibility and sound (*F*(1, 25) = 3.37, *p* = 0.08, eta^2^ = 0.12). No other main effects or interaction with those two factors were significant (all *p* > 0.10). When only investigating SWA in SWS this pattern remained. Please note however, that in this analysis 2 low suggestible subjects had to be excluded as they did not show SWS in one of the sessions. Examining beta power alone revealed a significant three-way interaction between sound, suggestibility and FCP (*F*(2, 50) = 6.55, *p* = 0.003, eta^2^ = 0.21). While in high suggestibles, beta power did not differ depending on sound (*p* > 0.80) or FCP* sound (*p* > 0.10), a main effect of sound (*F*(1, 12) = 5.46, *p* = 0.038, eta^2^ = 0.31) and its interaction with FCP (*F*(2, 24) = 4.56, *p* = 0.021, eta^2^ = 0.28) were found in low suggestibles. After listening to music, beta power was lower in low suggestibles (0.59 ± 0.09) than after text (0.67 ± 0.08). This difference was most pronounced in frontal electrodes (*t*(12) = −2.55, *p* = 0.026)) and central electrodes (*t*(12) = −2.26, *p* = 0.043) and not significant in parietal area (*t*(12) = −1.99, *p* = 0.070)).

We did neither find effects of sound in theta nor in alpha power (all *p* > 0.20).

Interestingly, power in the sigma band was reduced after music (1.19 ± 0.09) compared to the text condition (1.26 ± 0.10), *F*(1, 25) = 6.06, *p* = 0.02, eta^2^ = 0.20. The interaction between sound and suggestibility was a trend (*F*(1, 25) = 3.90, *p* = 0.06, eta^2^ = 0.14) and further specified by the three-way interaction between sound, FCP and suggestibility (*F*(2, 50) = 6.20, *p* = 0.004, eta^2^ = 0.20). In low suggestibles, sigma band power was higher in frontal and central electrodes after text compared to music (*t*(12) = −2.69, *p* = 0.02 and *t*(12) = −2.31, *p* = 0.04, respectively), while in high suggestibles, all direct comparisons were non-significant (all *p* > 0.20), see Fig. 2, lower panel. All other effects were non-significant (all *p* > 0.20). Due to significant effects in the power range of spindle activity, we analyzed number and density of spindles during NREM sleep. All effects were however non-significant (all *p* > 0.20, see Table 1). When restricting the analysis to frontal slow spindles, low suggestibles had descriptively reduced spindle density after music listening (3.02 ± 0.49) as compared to the control text (3.37 ± 0.60). However, the interaction between sound and suggestibility was not significant (*p* > 0.20). Also no significant main effects occurred (*p* > 0.07, see Table 1). The number of slow spindles also revealed no significant results (*p* > 0.50). All effects of density and number of parietal fast spindles were *p* > 0.50.

### Cardiac responses and poweranalyses during listening

As low suggestible participants profited more from relaxing music, it might have been possible that music induced stronger responses on the autonomic and central nervous system already during the listening period. In the ANOVA with sound and suggestibility on heart rate during listening, the interaction was not significant (*p* > 0.80). Also, neither the main effect of sound nor suggestibility reached significance (both *p* > 0.60). Similarly, during subsequent sleep, heart rate did neither differ depending on sound (*p* = 0.44), suggestibility (*p* > 0.70) nor sound * suggestibility (*p* > 0.60).

With respect to the power analysis of EEG activity during listening, we restricted our analysis to epochs during listening, in which the subjects were awake. Thus, all epochs containing stage N1 or stage N2 were excluded.

In this analysis, we observed a significant three-way interaction between sound, suggestibility and hemisphere for the SWA/beta ratio (*F*(1, 25) = 4.79, *p* = 0.038, eta^2^ = 0.16). In low suggestibles, the main effect of sound and all its interactions were *p* > 0.08 with non-significantly higher values during music than sound. In high suggestibles all follow-up tests were *p* > 0.20. The same pattern was true for the four-way interaction with FCP (*F*(2, 50) = 4.21, *p* = 0.020, eta^2^ = 0.14) and its follow-up tests (all *p* > 0.06 in low and *p* > 0.30 in high suggestibles). All main effects and interactions with sound or suggestibility were *p* ≥ 0.09 in the alpha and *p* > 0.10 in the theta and sigma band (see the Supplementary Material for the results of the power analyses of the entire listening period).

### Cognitive tasks

To investigate effects of altered sleep pattern on post-nap cognitive performance, we included vigilance task after sleep. Here, performance was equal in both groups and after both conditions concerning reaction times, number of reactions and error rate (*p* > 0.09). Thus, attentional processes after sleep did not differ dependent on condition.

Also, we measured memory consolidation across sleep by presenting paired associate learning task (PAL) before and after the nap. For this parameter, a main effect of sound (*F*(1, 24) = 4.45, *p* = 0.05, eta^2^ = 0.16) demonstrated that maintenance of memory performance across sleep was higher after listening to the text (99.89% ± 1.24) than across sleep after listening to music (94.95% ± 1.61). The other effects were *p* > 0.10. PAL performance across sleep with presleep memory set to 100% were 96.00 ± 1.87% after music and 101.25 ± 2.09% after text in low suggestibles and 94.04 ± 2.56% after music and 98.72 ± 1.46% after text in high suggestibles. Both differences were *p* > 0.10. The difference between PAL performance across nap after listening to music vs. text did not correlate with the difference in any sleep stage (all *p* > 0.15, uncorrected) nor subjective sleep quality (*p* > 0.20), nor the difference in any power band (all *p* > 0.10, uncorrected).

## Discussion

Here we tested if listening to relaxing music before a nap helps improving subjective and objective sleep quality of a nap in young healthy females. Our results showed a generally improving effect of listening to music on subjectively rated sleep quality. Subjects reported better sleep quality after having listened to music compared to listening to a control text. This is in line with previous studies showing beneficial effects of music on sleep ratings^14,18^. While subjective sleep is highly relevant for experienced wellbeing and the diagnosis of for instance insomnia, it does not necessarily correspond to objective sleep measures. We hence additionally measured sleep with polysomnography and compared sleep patterns after listening to music to sleep patterns after listening to a control text. Objectively, subjects spent less time in the sleep/wake transition phase N1 after music compared to the text condition. More fine-grained analyses showed that power in the sigma band (11–15 Hz) was also reduced during NREM sleep in the nap after listening to music versus listening to a text. Thus, overall, it can be concluded that listening to relaxing music before a nap can reduce time spent in N1 and the power of high frequency bands.

As we have seen in previous studies on the effect of hypnotic suggestions on a nap in healthy young and older females, it was important to consider subjects’ suggestibility. This measure indicates how sensitive subjects react to suggestions given externally. Concerning hypnosis, only those who were responsive for suggestions benefitted from the verbal, hypnotic intervention. To investigate whether suggestibility is also a relevant factor when testing the effect of music on sleep, we included this additional factor. We found that low suggestible subjects benefited from an increase in the amount of SWS of about 46% compared to their sleep after listening to the text. Whether this increase was to the detriment of the amount of REM sleep cannot be answered in this study due to a very limited amount of subjects reaching REM sleep at all during this nap. Additionally, as this was only a nap study we cannot make substantiated statements concerning REM sleep which usually takes place late in a sleep epoch. To confirm a possible trade-off between SWS and REM sleep the study should follow a nighttime design.

Also in a more fine grained analysis of oscillatory power bands during sleep, the level suggestibility significantly altered the effects of music. As studies have shown that insomniacs often experience low sleep quality when a high amount of high frequency penetrates their sleep^33,34^, we expected reduced high and increased low frequency power after listening to the relaxing music before sleep. A ratio quantifying the proportion of high and low frequency power in the NREM signal was calculated by dividing SWA power by beta power. Thus, higher values indicate a higher proportion of low compared to high frequencies in the signal. Low values had been referred to as an indication of worse sleep quality^32,34^ and less restorative sleep^35^. Here we found higher SWA/beta ratios during sleep after listening to music than after listening to the text in low suggestible subjects. No changes were observed in high suggestible participants. These results using objective sleep data suggest that low suggestibles might have experienced a more restorative sleep after listening to music.

In spite of more restorative sleep, sigma power in frontal and central brain regions during sleep was significantly reduced in low suggestibles after listening to music. However, we did not observe a reduction in sleep spindles in this condition, in spite of the reduction in sigma power. Previous studies have reported changes in spindle density without changes in sigma power (e.g.^36^), while here we report the opposite case. A possible explanation is that music reduces more long-lasting sigma power in low suggestible which might be unrelated to discrete sleep spindles (lasting maximal 3 seconds).

Interestingly, memory consolidation across the sleep interval was significantly reduced after listening to music as compared to the control text in the entire sample. The reduction in memory is particularly puzzling, because listening to non-verbal music after learning should induce less interference than the verbal control text. As SWS has been implicated in processes of memory consolidation during sleep^37,38^, increases in SWS and SWA /beta ratios in low suggestible participants should have led to improvements in declarative memory consolidation during sleep in low suggestible participants. It might be possible that the impairment in memory consolidation is related to the general reduction in sigma power in the music condition, as sleep spindles have suggested to be a critical factor for successful consolidation of memories during sleep^39^. However, as discrete sleep spindles were not altered by music in our study and changes in sigma power did not correlate with changes in memory performance, this possibility remains highly speculative and requires further examination. Note that memory consolidation was also not improved in spite of increases in SWS and SWA after hypnotic suggestions in high suggestibles in our previous studies^29,30^. In sum, low suggestibles benefitted more from relaxing music for their nap than high suggestibles. This is contrary to what we expected, as usually, a high level of suggestibility indicates more pronounced behavioral reactions to suggestions or expectations. Here we briefed the subjects about our expectations to find improved sleep after music compared to text. High suggestibles would have rather expected to be positively influenced by this information. However, also mere expectations without hypnotic induction did not lead to SWS increase in our nap study^29^. This suggests that high suggestibles show greater effects when the procedure is framed as hypnosis and a hypnotic induction actually takes place. On the contrary, low suggestibles seem to prefer non-verbal relaxation to hypnotic suggestions. This might result from a concern of being manipulated by direct and concrete instruction when being confronted with a spoken text. Under this assumption it could be even more effective to increase self-control by for instance letting subjects choose their own music. Trahan *et al*. (2018) discussed that familiar, self-chosen in contrast to given music might be less analgesic and anxiolytic^11^. These authors showed that particularly subjects with musical engagement use music to improve sleep. In our data, the distribution of subjects playing an instrument did not differ between high and low suggestible subjects. However, it might have possibly required a more precise measure of familiarity with music to test this assumption. Besides, effectiveness of a sleep-related intervention depends on whether sleep destabilization is triggered by psychological (i.e. mood, thoughts) or physical (i.e. arousal) factors. Being explicitly and verbally guided by hypnotic suggestions could thus be more expedient when rumination must be stopped while reducing physical tension could be better achieved by a more indirect and open form of relaxation intervention. Unfortunately, we did not measure those aspects in our sample to test group differences in this respect. Cardiac responses during the listening period and brain activity during wakeful listening remained unaffected. Probably, a combination of both, suggestions shaded with relaxing music could be beneficial for both needs and thus, all subjects could improve their sleep.

The importance of focusing on subjective and objective measures when investigating effects of any intervention on sleep was demonstrated by the fact that in neither of the groups the ratio or the amount of SWS was correlated with subjective sleep quality rating. Possibly separate mechanisms affect subjective and objective measures leading to converging results.

In sum, here we show that subjective sleep quality in young healthy females’ naps can be improved by relaxing music to some extent. Music reduced time spent in N1 and arousing high frequency power during following NREM sleep while leading to improved subjective sleep quality. This method seemed to be particularly beneficial for subjects low in suggestibility, although this conclusion should be tested critically in further studies and possible other confounding factors should be considered. Taken together, our results support the conclusion that music is a non-pharmacological, low-risk and low– cost tool to improve sleep on a subjective and objective level. To what extent this is applicable to sleep disturbances cannot yet be answered.

## Methods

### Subjects

Thirty-two healthy, right-handed women (19–35 years, mean age 23.81, SD = 4.28) participated. To avoid gender effects we excluded males. Three subjects had to be excluded due to lacking sleep in one of the sessions, reporting naps in the week before the experiment or heavy cough during the session. In two subjects suggestibility could not be measured, one subject did not correctly understand the declarative memory test and was excluded in that analysis. The final sample consisted of 27 subjects (aged 23.22 ± 3.85 years). Ten of them indicated to play an instrument (equally distributed between high and low suggestibles, Chi squared *p* = 0.29), four indicated to practice any kind of relaxation exercise. The subjects were German natives or had advanced German skills. They did neither regularly take naps, nor suffer from a diagnosed sleep disorder nor consume drugs. Hormonal contraceptives were allowed. None did shift work or intercontinental flights within 6 weeks before participation. For the experimental days they were asked to refrain from caffeine and alcohol, get up between 7 and 8 a.m. and to not do any sports until the session. Subjects received oral and written study information and gave their written informed consent before participation. They were paid 140 CHF for full participation. The Ethic Committee of the University of Zurich approved the study and the experiment was performed in accordance with the existing regulations.

### Procedure

The study consisted of four sessions. In the introduction group session, the study flow and its purpose were explained. We explicitly mentioned the expectation that music should improve sleep quality. Besides, questionnaires assessed sleep quality, demographic data and suggestibility. In the second session, subjects took a nap in the sleep laboratory to become familiar with the setup and sleep environment. The sleep diary was handed out to record sleep behavior in the week before the first experimental session. The experimental sessions took place at the same day of the week, separated by one week and started at 1 p.m. After performing the paired associate task and the finger tapping task, subjects went to bed. Depending on randomization, either music or the control text was presented via loudspeakers from the bedside cabinet when subjects lay in bed directly after switching the lights off. Participants were allowed to fall asleep at any time, but asked to listen to the sound. Listening and nap were recorded for a total of 90 minutes with polysomnography (PSG). Subjects were awakened 90 minutes after switching the lights off and asked to fill out the sleep quality questionnaire and perform again on the memory tasks. After each session, the sleep diary for the upcoming week was handed out.

### Material

#### Audio recordings

Music: Before the experimental naps, subjects either listened to a music tape or a spoken text. The selection of the musical piece was performed in a pilot study: Five 15 minutes-pieces of music, composed to induce sleep, were selected according to internet evaluations (google, youtube, amazon). Fifteen subjects listened to this selection before falling asleep and rated the tapes the next morning. According to subjectively reported enhancement of sleep quality, shortened sleep latency and better recovery, the best rated tape was chosen for the study. It was a piece composed by Dr. Lee Bartel for promoting sleep, called “Drifting into Delta”track^2,31^. It is supposed to induce deep sleep through its frequencies of 0.01–2 Hz, based on treatises on the influence of auditory pulsing of 0.25 to 2 Hz frequencies on oscillatory coherence^40^. Please find the spectrogram displaying the contained frequencies in the Supplementary Material. The series “Music to Promote Sleep” out of which we took this piece was tested before in patients suffering from fibromyalgia and showed positive effects on subjective sleep quality^41^.

Text: The text in the control condition was taken from Cordi *et al*.^29^ and was a documentary about mineral deposits. A text was chosen as control to exclude a simple effect of listening. It was spoken by a male voice in normal speed and intonation.

Both tapes played for about 14 minutes. They were presented at a volume of 45–50 dB through loudspeakers before falling asleep. The order was determined randomly across subjects. Before playing the sound we instructed the subjects to relax and to get themselves into the tape, trying to let pass any thoughts that might come up and to not stuck on them. Subjects were allowed to fall asleep whenever possible.

#### Questionnaires

Standardized Sleep Inventory for the Assessment of Sleep – Revised Version, SFA – R: It assesses subjectively reported sleep behavior within the last sleep period (adapted to a nap here) on several dimensions^42^. As a measure for subjective sleep quality we analyzed the mean of an item asking to rate the previous sleep on 7 adjectives. We excluded the adjective “extensive” as the nap was limited in time by protocol. Higher values indicate better ratings. Data of one subject is missing.

Pittsburg Sleep Quality Index, PSQI: The PSQI measures general subjective sleep quality within the last month^43^. The global score ranges from 0 to 21, while 5 is the cutoff value for sleep difficulties. Overall, the sample scored 4.41 ± 2.26 ranging from 1 to 10 (5 subjects scoring >5). High and low suggestible subjects did not differ on this score (*p* = 0.83).

Sleep diary: To control sleep behavior one week before each of the experimental sessions, a sleep diary was used. We used the information about the wake up time to verify that subjects got up between 7 and 8 a.m. on experimental days.

Harvard Group Scale of Hypnotic Susceptibility, Form A, HGSHS: A: This is a widely used, standardized tool to determine hypnotic suggestibility^44^. After listening to a tape with a recorded hypnosis, subjects are confronted with a questionnaire on their experiences during listening. According to their rating about how strongly they had reacted to the given suggestions, they were grouped as high (score 7 or higher, mean = 8.50 ± SD = 1.23, n = 14) or low suggestible (scores 0–6, mean = 4.85 ± 1.21, n = 13). The groups differed significantly on those means, *t*(25) = −7.78, *p* < 0.001, but not on age (*p* > 0.50).

#### Memory measures

Word pair associate learning task, PAL: In this episodic memory task, subjects are asked to remember as many of the presented word pairs as possible for a later cued recall^45^. The words are presented consecutively in EPrime in black font on a white computer screen. After a 500 ms fixation cross, the first word of the pair was presented for 1000 ms, followed by a blank interval of 200 ms separating it from the according second word of the pair. Another blank interval of 500 ms was displayed before the next fixation cross separating the previous from the following word pair. During cued recall, the first word of the pair was displayed until the subject remembered the according second word or indicated that it was forgotten. No feedback was given. The order during recall was different from the order during learning, but stable across subjects. Recall was tested directly before the nap and afterwards in the same order. Memory performance was defined as the number of correctly recalled words after sleep relative to the presleep performance which was set to 100%.

The procedural memory task and its results are reported in the Supplementary Material.

Psychomotor vigilance task (PVT): To overcome sleep inertia and to control for possible attentional differences before the memory tests after the nap, a vigilance task was applied. Subjects were confronted with a black screen on which a timer started to count upwards at unforeseen times. As soon as this was recognized, subjects were asked to press the space bar. Error rate and reaction time were analyzed.

#### Sleep recordings, scoring and EEG data processing

Sleep was measured with the Geodesic EEG System 400 series (Electrical Geodesics, Inc.) including high density electroencephalogram (EEG) with 128 electrodes, electromyogram (EMG), electrooculogram (EOG) and electrocardiogram (ECG). Data was collected with a sampling rate of 500 Hz, impedances were kept below 50 kΩ. Data was filtered and scored according to the criteria of the American Association of Sleep Medicine (AASM) (i.e., 0.1 Hz lowpassfilter, 35 Hz highpassfilter). Three coworkers blind to condition scored the data at the electrodes F4, C4, P4, O2, HEOG, VEOG and EMG. In case of disagreement, a forth blind scorer was consulted.

Power analyses were based on Fast Fourier Transformations (FFT). During data preprocessing we excluded the electrodes on the outer edge of the EEG cap (i.e. electrode numbers 17, 48, 49, 56, 63, 68, 73, 81, 88, 94, 99, 107, 113, 119, 125), filtered the data between 0.1 and 50 Hz and applied a 50 Hz Notch Filter. We re-referenced data to the average of both mastoids. The signal that was recorded while the music and the control file were presented was segmented into epochs of 4096 data points (~8 seconds) with an overlap of 409 points to account for the later applied Hamming-window of 10% and semi-automatically corrected for artefacts within the wake periods in the 14 minutes in which subjects listened to the tape. The same was done for segments scored as NREM sleep. Afterwards, the FFT was calculated with a Hamming-window of 10% and a resolution of 0.2 Hz. From this analysis, we exported the power in the respective areas (muV*Hz) for slow-wave activity, SWA (0.5–4.5 Hz), theta (4.5–8 Hz), alpha (8–11 Hz), sigma (11–15 Hz), beta (15–30 Hz) and the total power (0.5–50 Hz). Similarly to Krystal^32^ and Maes *et al*.^33^ we focused on the ratio between low and high frequencies during NREM sleep as an indicator for restorative sleep. SWA power was divided by beta power and hence higher values would indicate slowing of the EEG activity.

For the analyses on spindle density, we selected the derivations Fz, Cz and Pz for the analysis of the spindles across all sleep stages. On those, we performed a frequency extraction for frequency power of 11–15 Hz and added Spindle on and off markers to identify its on- and offset. Before automatic artifact correction detecting a maximal allowed difference of 200 muV within 200 ms in the EMG channel, we segmented the data into 1024 data point wide epochs (~2 seconds). Spindle events were counted for which the power signal exceeded a fixed threshold (±10 muV) for an interval lasting 0.5–3 sec. We extracted the amount of detected spindles (=spindle count) in those epochs separately for each sleep stage and separately for fast (13–15 Hz) and slow spindles (11–13 Hz). We then calculated spindle density per 1 minute within each sleep stage, also within NREM sleep (N2 + N3).

Heart rate: We analyzed electrocardial data with Kubios HRV Version 3.1 2. We first ran an automatic artifact correction on the unfiltered data that eliminated ectopic beats and artifacts based in dRR series. Afterwards, we excluded further visually detectable artifacts resulting from e.g. movements. Mean heart rate (HR) was measured for the time in which the subjects listened to the tapes and the following sleep episode separately. We could include all except 2 subjects for who the signal was too bad in one of the two sessions (1 low, 1 high suggestible).

### Statistical analysis

The study followed a crossover design with a within-subjects comparison of the two naps. Data was analyzed using SPSS 23. The repeated measures analysis of variance (ANOVA) included the within subjects factor “sound” (music versus text) and the between subjects factor “suggestibility” (high versus low). When the Mauchly-Test was significant, we adapted values with Greenhouse-Geisser. We also adapted values when the Levene Test indicated uneven variances. Only the significant main effects and interactions were further investigated using paired samples t-tests according to Fisher’s protected LSD test. The level of significance was set to *p* = 0.05.

## Supplementary information

Supplementary Material

## Data Availability

The datasets generated during and/or analysed during the current study are available from the corresponding author on reasonable request.
