# Supplementary material for: Effects of Relaxing Music on Healthy Sleep
Source: Sci Rep. 2019 Jun 24;9:9079. doi: 10.1038/s41598-019-45608-y (PMC6591240; doi:10.1038/s41598-019-45608-y)
Supplement: Supplementary file 1 — Supplementary Material [file 41598_2019_45608_MOESM1_ESM.pdf]

EFFECTS OF RELAXING MUSIC ON HEALTHY SLEEP

- Supplementary Material -

Maren Jasmin Cordi[1,2], Sandra Ackermann[1] and Björn Rasch[1,2]*

1 University of Fribourg, Department of Psychology, Division of Cognitive Biopsychology and Methods, Fribourg, Switzerland
2 Sleep & Health Zürich, University of Zurich, Zurich, Switzerland

*Corresponding author:

Björn Rasch, University of Fribourg, Department of Psychology, Division of Cognitive Biopsychology and Methods, Rue P.-A. de Faucigny 2, 1700 Fribourg, Switzerland, Phone: +41 26 300,76 37 Fax: +41 26 300 97 12

Email: bjoern.rasch@unifr.ch

1. Methods

Procedural finger tapping task [1]. Subjects were asked to type a five-element finger sequence (4-1-3-2-4 or 4-2-3-1-4) displayed on the screen with their non-dominant hand as fast and accurately as possible on the keyboard. Learning consisted of nine 30-second blocks while the same sequence was continuously displayed. 30 second breaks interrupted the blocks. The mean of the last three trials was used as presleep performance. The recall after sleep contained only three trials, which were averaged for postsleep performance. After each block, feedback on the number of completed sequences and error rate was provided. Procedural memory performance in terms of speed and accuracy were calculated as relative performance after sleep compared to presleep which was set to 100%.

Audio recording: Music. We display the frequencies contained in the music piece in Figures S1 and S2 for the whole spectrum (0 – 16000 Hz) and the lower frequencies (0 – 2000 Hz).

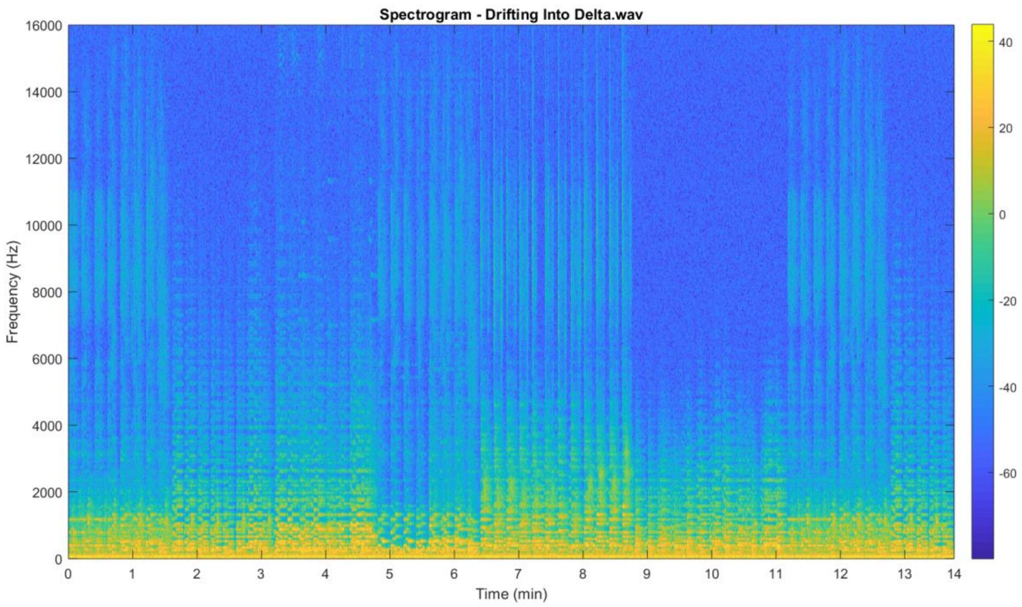

Figure S1. Spectrogram displaying the power of frequencies of 0 – 16000 Hz across the 14 minutes of the piece “Drifting into Delta”.

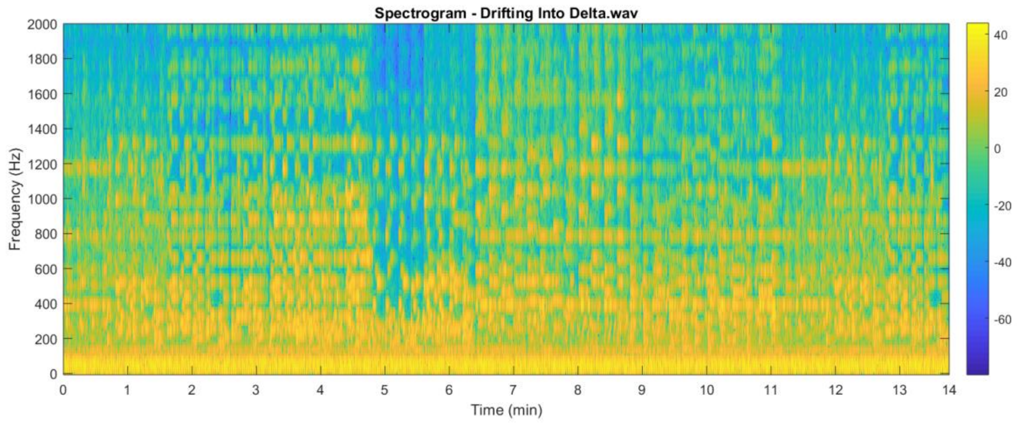

Figure S2. Spectrogram displaying the power of frequencies of 0 – 2000 Hz across the 14 minutes of the piece “Drifting into Delta”.

2. Results

2.1 Poweranalyses during listening

In the power analysis of EEG activity during the whole period of listening including wakefulness and sleep epochs, we observed an increased SWA/beta ratio during listening of the music (11.45 ± 1.90) as compared to the text (8.16 ± 0.79) independent of suggestibility (main effect of sound $F(1, 25)$ =

4.24, p = .049, eta^2=.15). The effect was most pronounced in frontal electrodes ($t(26)$ = 2.44, p = .022), whereas no significant differences occurred at central or parietal recording sites (p = .10 and p = .11, respectively, interaction sound * FCP $F(2, 50)$ = 6.40, p = .003, eta^2= .20). However, low vs. high suggestible participants did not differ in the SWA/beta ratio during listening of music (all other interactions with suggestibility and sound $p \geq .06$).

For theta power, all effects including sound were $p > .20$.The interaction between FCP and suggestibility ($F(2, 50)$ = 4.16, p = .02, eta = .14) and FCP * suggestibility * hemisphere ($F(2, 50)$ = 4.07, p = .02, eta^2 = .14) were further reflections of the main effect of suggestibility that we found in this frequency band ($F(1, 25)$ = 4.80, p = .038, eta^2 = .16). Low suggestibles had higher theta power (3.26 ± 0.64) than low suggestibles (1.73 ± 0.31), which was pronounced in frontal electrodes ($t(25)$ = 2.53, p = .02), decreasing through central ($t(26)$ = 2.20, p = .037) to parietal electrodes ($t(26)$ = 1.75, p = .09). Other effects in theta power were $p > .20$.

For alpha and sigma power, we observed differential responses during listening of the music for low vs. higher suggestible (significant interaction between suggestibility and sound, alpha: $F(1, 25)$ = 4.55, p = .04, eta^2 = .15; sigma: $F(1, 25)$ = 5.02, p = .034, eta^2 = .17). In spite of the significant interaction, post-hoc tests revealed only statistical trends. Alpha power tended to be reduced during listening to music in low suggestible subjects (4.07 ± 1.17 vs. 5.07 ± 1.36), $t(12)$ = 2.05. p = .06, but not in high suggestibles (p = .69). Main effect of sound ($F(1, 25)$ = 3.46, p = .075, eta^2 = .12) and suggestibility ($F(1, 25)$ = 3.91, p = .06, eta^2 = .14) were a trend. All other effects were $p > .30$. Analyzing the first 14 minutes of the adaptation nap showed that alpha power without listening (3.48 ± 0.76) was between text (3.64 ± 0.82) and music (3.14 ± 0.68) condition. Sigma power during listening to music was not significant in low suggestibles (p = .26), but tended to be increased in high suggestibles compared to text (p = .07). Low suggestibles generally had a higher level of sigma power (1.13 ± 0.18) than high suggestibles (0.75 ± 0.06), $F(1, 25)$ = 4.28, p = .05, eta^2 = .15. The effect of sound was dependent on FCP ($F(2, 50)$ = 4.39, p = .017, eta^2 = .15), which was however not confirmed in post-hoc analyses (all $p > .20$). All other effects with sound or suggestibility were $p > .30$.

As the SWA/beta ratio was generally increased during listening to music before sleep in the entire sample, we tested whether this change during music listening (as compared to the control text) predicted the reported general effects of music vs. text on subjective sleep quality and N1 sleep (see Table 1) as well as sigma power (see previous section). However, all three correlations were not significant (all $p > .20$). In addition, we observed a specific increase in alpha power during listening of music in low suggestible participants. Thus, we tested whether alpha power during listening of music predicted the specific effects of music on objective sleep variables reported in low suggestible in

%SWS (see Table 1) and SWA/beta power ratio during NREM sleep. Again, none of these correlations was significant (all $p > .30$).

Thus, differences in frequency power during listening of music vs. the control text cannot explain the effects of music during later sleep, neither in the entire sample nor in low suggestibles.

2.2 Procedural memory task

For the procedural task all effects were non-significant ($p > .40$).
